# Supplementary material for: Mapping the aggregate g-ratio of white matter tracts using multi-modal MRI
Source: Imaging Neurosci (Camb). 2025 Jun 18;3:IMAG.a.49. doi: 10.1162/IMAG.a.49 (PMC12319806; doi:10.1162/IMAG.a.49)
Supplement: Supplementary Material [file imag.a.49_supp.pdf]

Mapping the aggregate g-ratio of white matter tracts using multi-modal MRI

Supplementary Material

Wen Da Lu[1,2], Mark C. Nelson[2,3], Ilana R. Leppert[2], Jennifer S.W. Campbell [2], Simona Schiavi[4], G. Bruce Pike [5], Christopher D. Rowley[6], Alessandro Daducci [4], Christine L. Tardif[1,2,3]

1. Department of Biomedical Engineering, McGill University, Montreal, QC, Canada
2. McConnell Brain Imaging Centre, Montreal Neurological Institute and Hospital, Montreal, QC, Canada
3. Department of Neurology and Neurosurgery, McGill University, Montreal, QC, Canada
4. Department of Computer Science, University of Verona, Verona, Italy
5. Hotchkiss Brain Institute, Department of Radiology, and Department of Clinical Neuroscience, University of Calgary, Calgary, AB, Canada
6. Department of Physics and Astronomy, McMaster University, Hamilton, ON, Canada

Corresponding authors:

Wen Da Lu, wen.d.lu@mail.mcgill.ca

Christine L. Tardif, christine.tardif@mcgill.ca

Two methods of using COMMIT to extract the true intra-axonal volume of a tract:

In the main manuscript, we used COMMIT to compute the true-intra axonal volume (AV) of a tract. An alternative method involves generating a voxel-wise AV fraction map from the NODDI ICVF map and calculating the tract AV using the COMMIT bundle approach (Schiavi et al., 2022). However, this method is less powerful than COMMIT (Daducci et al., 2014) which leverages the full diffusion-weighted dataset across multiple gradient directions and b-values to better disentangle the signal contribution of each streamline. In contrast, standard 3D images provide only voxel-level spatial information. To evaluate the performance of both techniques, we compared their fits using a NRMSE map. To ensure a fair comparison, we eroded the NRMSE map to exclude voxels affected by partial voluming with gray matter, as these voxels tend to have abnormally high NRMSE due to how NODDI models gray matter. Using COMMIT with the 4D diffusion dataset, the mean NRMSE across all scans was 4.9%. In comparison, the bundle approach applied to the ICVF map from NODDI yielded a mean NRMSE of 11.2%. For reference, the mean NRMSE of the MVF map generated using the bundle approach was 8.3%, calculated at a native resolution of 1 mm isotropic.

Supplementary Figures:

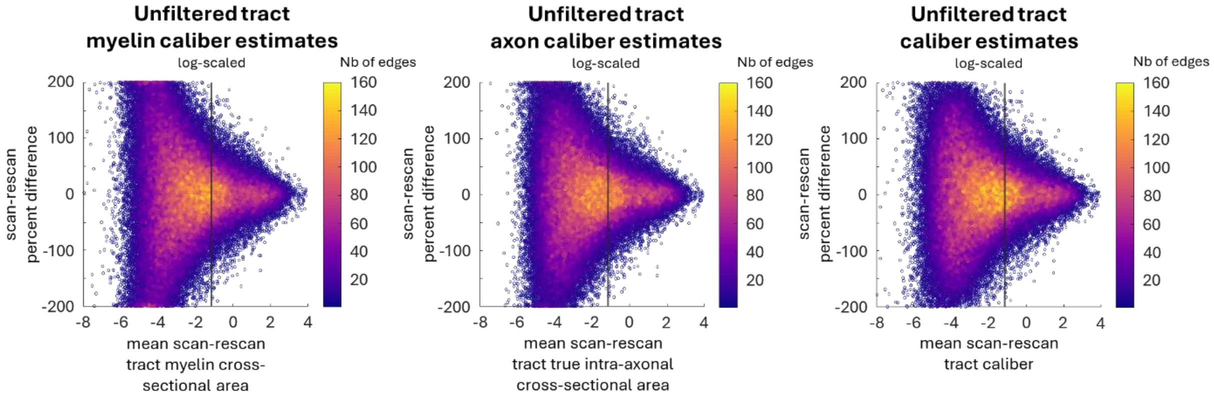

Figure S1: Percent scan-rescan difference as a function of the tract's total myelin cross-sectional area, total axonal cross-sectional area, and tract caliber, i.e. the sum of the myelin and axonal areas (log-scaled). The black vertical line indicates the 80% cutoff threshold used in the subsequent analyses. Larger tracts are more repeatable than for smaller caliber tracts.

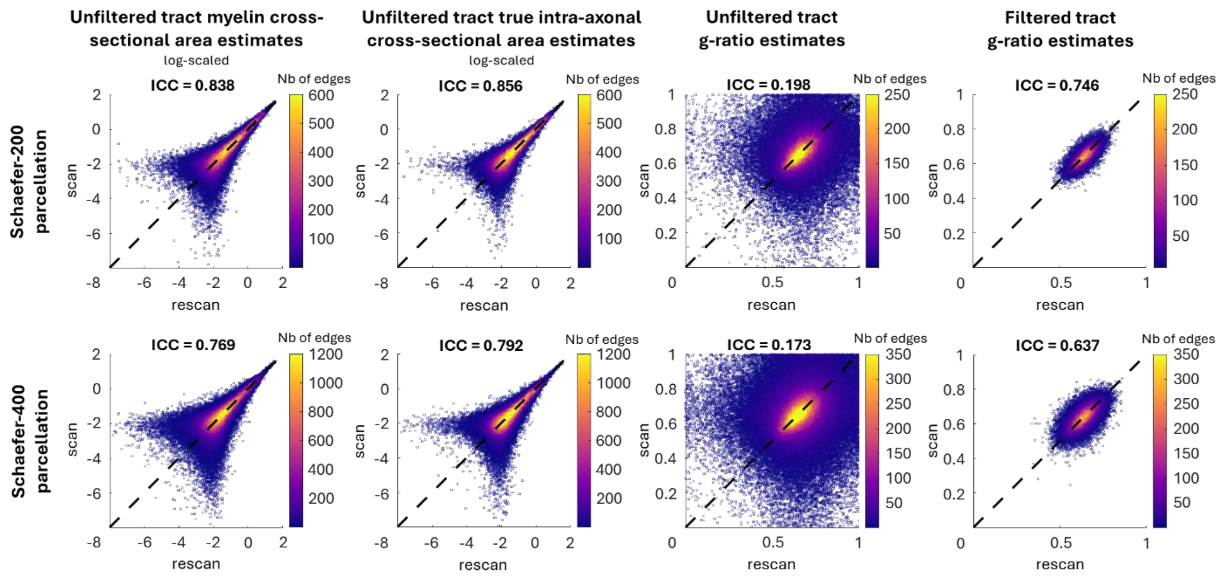

Figure S2: Impact of cortical parcellation resolution on scan-rescan repeatability of tract-specific g-ratio. The figure illustrates a tract's myelin caliber, axon caliber, unfiltered, and filtered g-ratio plotted against its rescan at two resolutions: Schaefer-200 (top row) and Schaefer-400 (bottom row). The correlation plot of myelin and true intra-axonal cross-sectional area has been log-scaled. The ICC for myelin cross-sectional area estimates at Schaefer-200 and-400 parcellations is 0.838 and 0.769, respectively. For the true intra-axonal cross-sectional area estimates, the ICC at Schaefer-200 and-400 parcellations is 0.856 and 0.792, respectively. The ICC for unfiltered g-ratio estimates at Schaefer-200 and-400 parcellations is 0.198 and 0.173, respectively. Finally, the ICC for percentile and consensus filtered g-ratio estimates at Schaefer-200 and-400 parcellations is 0.746 and 0.637, respectively.

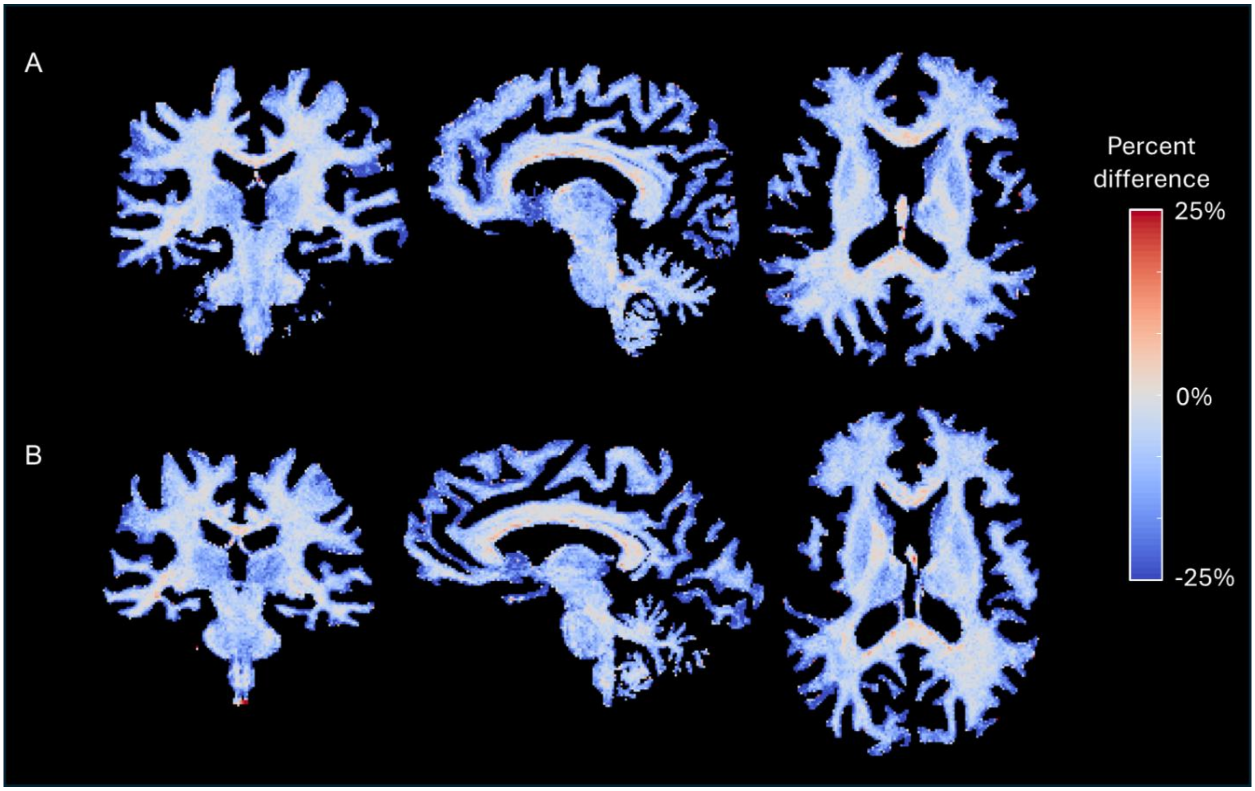

Figure S3: The absolute percent difference between the g-ratio map computed from COMMIT's AVF and MVF maps and the volumetric g-ratio calculated from NODDI for subjects (A and B). A negative percent difference indicates a lower g-ratio for the g-ratio map computed from the COMMIT framework. While the percentage difference in voxels along major white matter pathways is low, it increases near the cortex and in the subcortical regions. This suggests that shorter white matter tracts and tracts traversing subcortical regions are the primary drivers of the g-ratio differences between tract-specific and tractometry approaches.

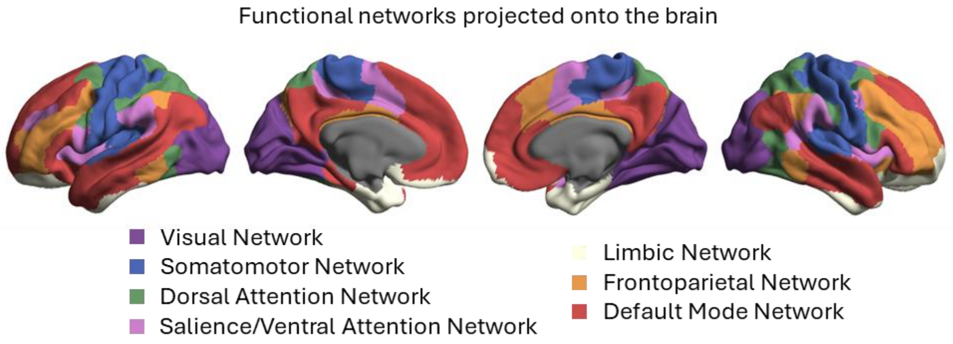

Figure S4: Cortical projections illustrating the functional network affiliation of each node in the Schaefer-200 parcellation. Each node is assigned to one of seven functional networks, providing a spatial representation of network organization across the cortex.

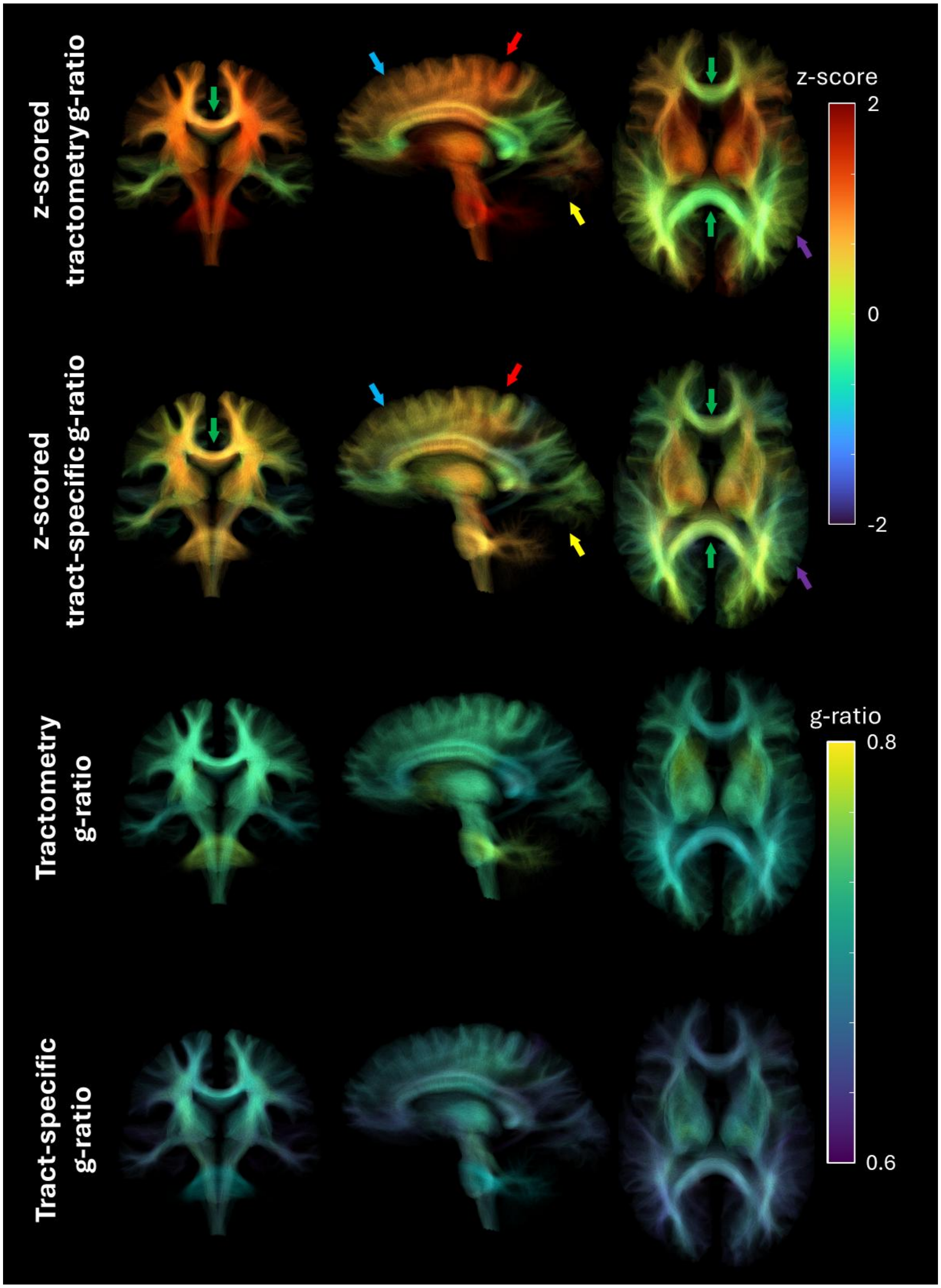

Figure S5: Streamlines spanning the entire brain of a participant. The top two rows are color-coded based on z-scored g-ratios, while the bottom two rows use g-ratio values. The arrows highlight regions of interest: red arrows point to the CST, yellow arrows point to the visual regions, and blue arrows indicate the frontal region, which show higher z-scored values in tractometry; purple arrows highlight the parietal region, and green arrows mark the corpus callosum, where both methods yield similar z-scored values.

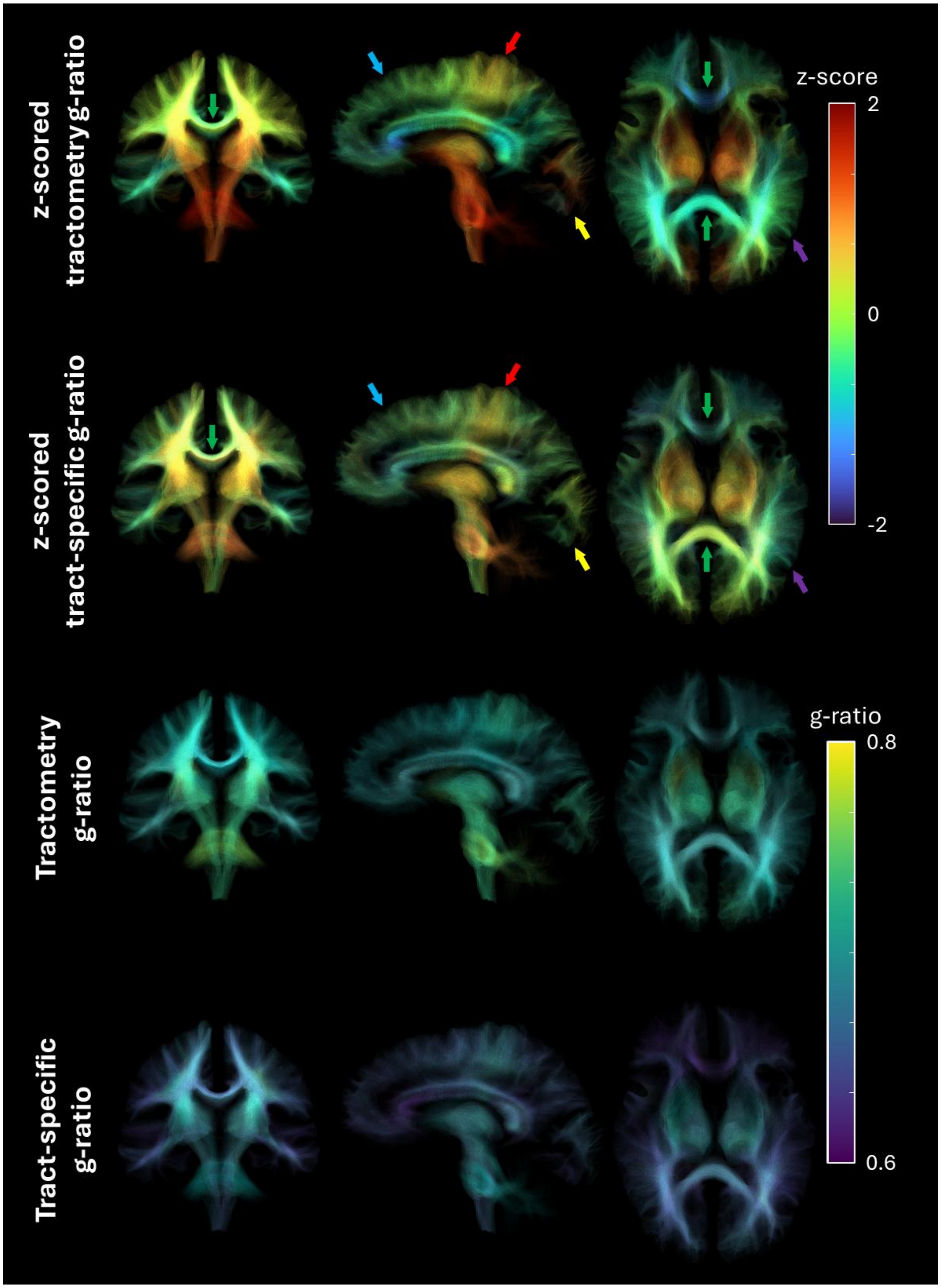

Figure S6: Streamlines spanning the entire brain of a second participant.

References:

Daducci, A., Dal Palù, A., Lemkaddem, A., & Thiran, J.-P. (2014). COMMIT: convex optimization modeling for microstructure informed tractography. *IEEE transactions on medical imaging*, *34*(1), 246-257.

Schiavi, S., Lu, P.-J., Weigel, M., Lutti, A., Jones, D. K., Kappos, L., Granziera, C., & Daducci, A. (2022). Bundle myelin fraction (BMF) mapping of different white matter connections using microstructure informed tractography. *NeuroImage*, *249*, 118922.
